# Salt stress causes cell wall damage in yeast cells lacking mitochondrial DNA

**DOI:** 10.15698/mic2014.01.131

**Published:** 2014-03-03

**Authors:** Qiuqiang Gao, Liang-Chun Liou, Qun Ren, Xiaoming Bao, Zhaojie Zhang

**Affiliations:** 1 State Key Laboratory of Bioreactor Engineering, East China University of Science and Technology, 130 Meilong Road, Shanghai 200237, China.; 2 Department of Zoology and Physiology, University of Wyoming, Laramie, WY 82071, USA.; 3 State Key Laboratory of Microbial Technology, Shandong University, Jinan, China.

**Keywords:** cell wall damage, reactive oxygen species, SCW11, salt stress, Saccharomyces cerevisiae

## Abstract

The yeast cell wall plays an important role in maintaining cell morphology, cell integrity and response to environmental stresses. Here, we report that salt stress causes cell wall damage in yeast cells lacking mitochondrial DNA (ρ^0^). Upon salt treatment, the cell wall is thickened, broken and becomes more sensitive to the cell wall-perturbing agent sodium dodecyl sulfate (SDS). Also, *SCW11* mRNA levels are elevated in ρ^0^ cells. Deletion of *SCW11* significantly decreases the sensitivity of ρ^0^ cells to SDS after salt treatment, while overexpression of *SCW11* results in higher sensitivity. In addition, salt stress in ρ^0^ cells induces high levels of reactive oxygen species (ROS), which further damages the cell wall, causing cells to become more sensitive towards the cell wall-perturbing agent.

## INTRODUCTION

The yeast *Saccharomyces cerevisiae* cell wall occupies about 15% of the total cell volume and plays important roles in maintaining cell shape, cell integrity and protection against environmental stresses 1. The yeast cell wall forms a microfibrillar network complex composed of glucan, mannoprotein and chitin. Glucan is mainly (80 to 90%) composed of β-1,3-glucan chains with some β-1,6-linked glucan branches. Glucan represents 50-60% of the cell wall mass and its main function is to maintain cell elasticity that can adapt to different physiological states (sporulation or budding) and various stress conditions, including exposure to cell wall-perturbing agents or hypo-osmotic shock 2,3.

Scw11p is a cell wall protein similar to glucanases, whose main function is to break down glucans 4,5. Whole-genome transcriptional analysis suggests that Scw11p is required for efficient cell separation following cytokinesis 6, and is also associated with cell wall metabolism 7. Mutation of *scw11 *suppresses the phenotype of Δscw4/Δscw10 double mutants, or Δscw4/Δscw10/Δbgl2 triple mutants, suggesting that Scw11p has an activity antagonistic to that of Scw4p, Scw10p and Bgl2p 8.

Reactive oxygen species (ROS) play a crucial role in the signal transduction pathways that regulate yeast cell death 9,10. Several studies have shown that ROS function as inducers of yeast apoptosis 11, while others have demonstrated that ROS play a crucial role as mediators of apoptosis 12,13. It has been shown that ROS accumulate in yeast cells after induction of apoptotic death by various stimuli, and are necessary and sufficient to induce an apoptotic phenotype in yeast 11,14,15,16.

Previously, we showed that yeast lacking mitochondrial DNA (ρ^0^) undergoes apoptosis upon salt stress and that cell death is mediated by cytochrome *c* 17. In this study, we further report that salt stress causes cell wall damage (CWD) in ρ^0^ cells and that this damage is related to elevated levels of *SCW11* and salt stress-induced ROS.

## RESULTS AND DISCUSSION

### Salt stress causes cell wall damage (CWD) in ρ^0^ cells

**Figure 1:**
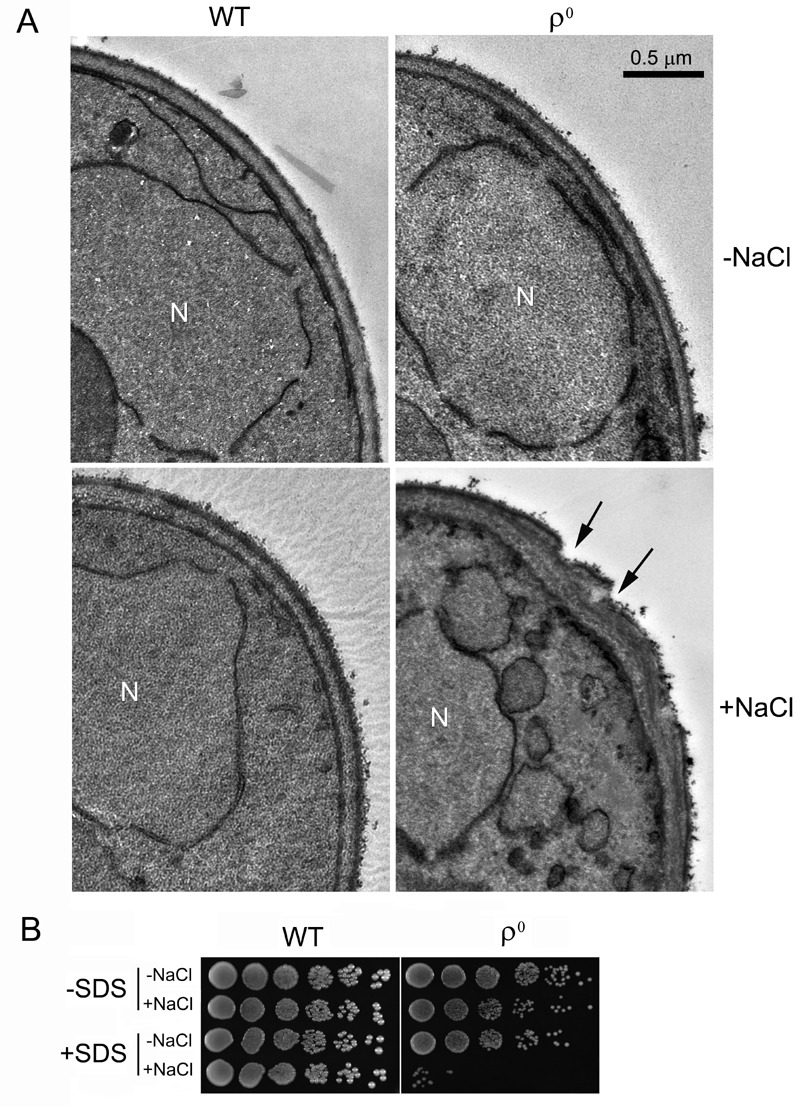
FIGURE 1: **(A)** TEM images of WT and ρ^0^ cells growing in YPD medium with or without treatment of 0.6 M NaCl for 1 hr. A normal cell wall structure was observed in WT either with or without NaCl. The cell wall structure was damaged in ρ^0^ cells upon NaCl treatment. Arrows indicate damaged cell wall. N = nucleus. **(B)** ρ^0^ cells are hypersensitive to SDS after NaCl treatment. Cells were first grown in YPD liquid media containing 0.6 M NaCl for 1 hr, then washed, treated with 0.1% SDS for 0.5 hr, spotted on YPD plates using 4 μl of 1:5 serial dilutions and incubated for 2-3 days at 30^ο^C.

We previously reported that ρ^0^ cells were sensitive to salt stress and consequently underwent apoptotic cell death 17. In the current study, our TEM analysis revealed that salt stress caused an abnormal cell wall structure in ρ^0^ cells. When treated with NaCl, the cell wall of more than 50% of the ρ^0^ cells became thicker and uneven, compared to ρ^0^ cells without NaCl treatment or to the wild type (WT). Certain areas of the cell wall also appeared abrupt, or damaged (Fig. 1A). To further examine possible salt stress effects on the cell wall, we tested the sensitivity of ρ^0^ cells towards sodium dodecyl sulfate (SDS), a typical cell wall-perturbing agent, before and after salt stress. As shown in Fig. 1B, no apparent change was observed in WT cells when treated with NaCl, SDS or both. ρ^0 ^cells, however, were hypersensitive to SDS after salt stress compared to cells treated with salt or SDS alone, further suggesting that the cell wall was damaged by the salt stress.

### SCW11 is involved in CWD of ρ^0^ cells under salt stress 

**Figure 2:**
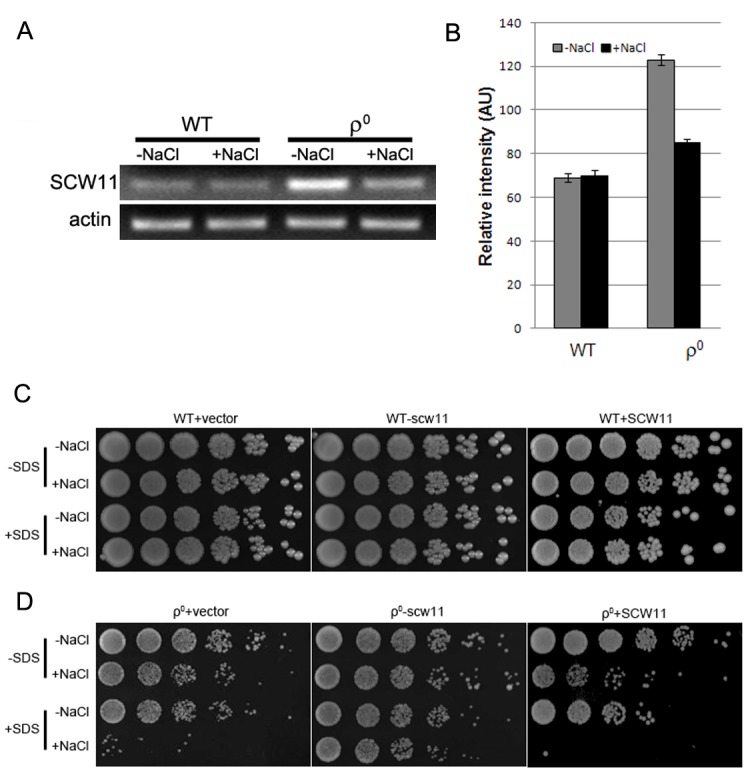
FIGURE 2: **(A)** RT-PCR analysis of ρ^0 ^cells showing *SCW11* expression upregulated compared to WT. *SCW11* expression in ρ^0 ^cells was decreased when treated with 0.6 M NaCl for 30 min, but still higher than in WT cells. **(B)** Quantitative analysis of the RT-PCR bands from three independent experiments. (C) and (D) The cell wall gene *SCW11 *is involved in salt stress-induced cell death in ρ^0 ^cells. **(C)** When treated with NaCl, SDS, or both, no apparent change is observed in the growth of WT cells when *SCW11* is either deleted or overexpressed. **(D)** Deletion of *SCW11* in ρ^0^ cells (ρ^0^-scw11) increases their resistance to salt; SCW11 overexpression (ρ^0^+SCW11) causes cells to become more sensitive to SDS after NaCl treatment (0.6 M NaCl for 1 hr). The BG1805 empty vector (WT/ρ^0^+vector) was used as a negative control. Cells were spotted on YPG using 4 μl of 1:5 serial dilutions and cultured at 30^ο^C for 3-4 days.

Scw11p is a cell wall protein with an endo-1,3-β-glucanase activity. It has been previously reported that the* SCW11 *gene is up-regulated in ρ^0 ^cells 18. However, it is still elusive how *SCW11* is involved in the salt stress response in ρ^0 ^cells. In this study, *SCW11* gene expression was first examined using RT-PCR with or without salt treatment. Consistent with microarray analyses 18, we showed that in the absence of NaCl, the *SCW11* expression level was significantly (P < 0.001) higher in ρ^0^ cells than in the WT cells. Under salt stress conditions, *SCW11* expression in ρ^0^ cells was decreased but still significantly higher (P < 0.05) than in WT cells (Fig. 2A, 2B).

To further assess the role of *SCW11* during salt stress response, *SCW11* was deleted or overexpressed in both WT and ρ^0 ^cells. As shown in Fig. 2C, deletion or overexpression of *SCW11* had no apparent effect on the WT. However, deletion of *SCW11* greatly enhanced the survival rate of ρ^0 ^cells, especially when the cells were treated with both NaCl and SDS. *SCW11 *overexpression caused ρ^0 ^cells to become more sensitive towards salt stress and SDS (Fig. 2D). These results suggest that, under salt stress conditions, *SCW11 *is involved in cell wall organization and its downregulation in ρ^0 ^cells (Fig. 2A) is part of a protection mechanism against salt stress.

The yeast cell wall is a dynamic structure that can adapt to different physiological states and various stress conditions 2,3. The endo-β-1,3-glucanase is an abundant protein in the yeast cell wall making it rigid by inserting intra-chain linkages into 1,3-β-glucan 19. Overexpression of the endo-β-1,3-glucanase Bgl2p has been shown to lead to defects in the cell wall structure and to sensitivity towards osmotic stress 20. Similar to this result, we show that the *SCW11* gene, coding for a glucanase like-protein, is upregulated in ρ^0 ^cells, where it leads to a higher sensitivity towards SDS and salt stress.

### ROS is involved in CWD of ρ^0 ^cells under salt stress

**Figure 3:**
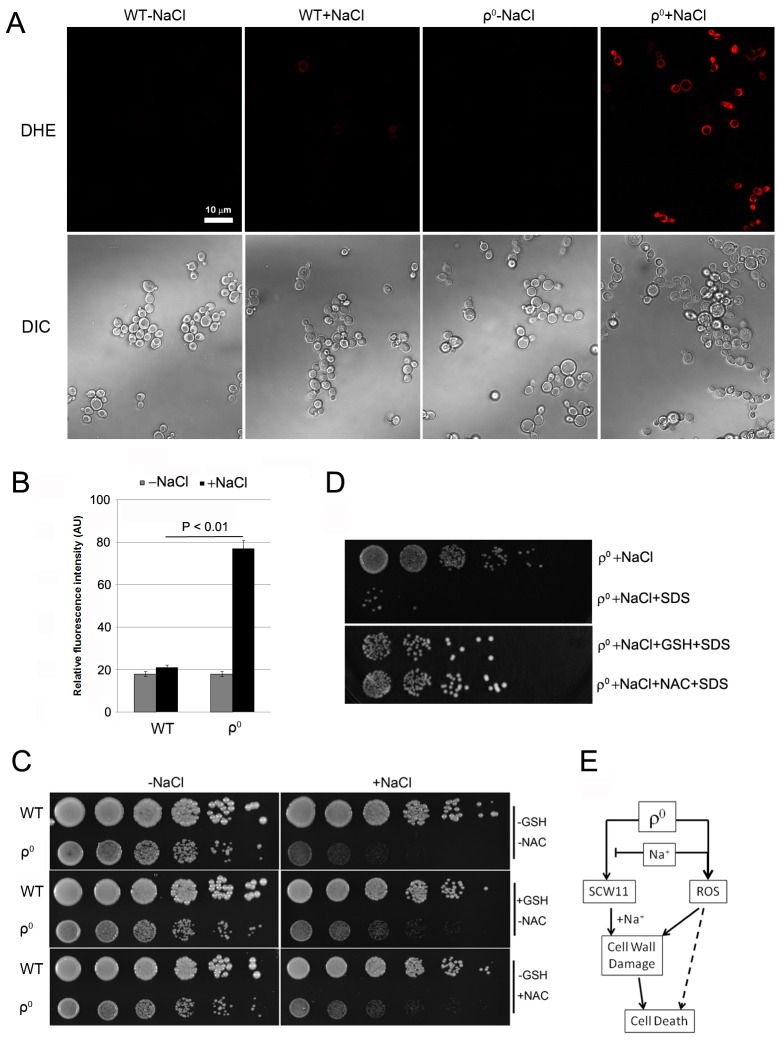
FIGURE 3: **(A)** and **(B)** Reactive oxygen species (ROS) production in the WT and ρ^0^ cells treated with 0.6 M NaCl for 15 min. **(A)** After salt stress, ρ^0^ cells displayed a higher fluorescence level than WT cells. Staining with 5 µM dihydroethidium (DHE) was used. **(B)** Quantification of ROS production. Relative fluorescence intensities were measured by the ImageJ software. Values presented are means of three independent experiments. About 300 cells were measured during each experiment. **(C)** Antioxidant GSH or NAC slightly decreases the salt sensitivity of ρ^0^ cells. Log phase cells were spotted directly onto YPD plates containing 0.6 M NaCl and GSH (10 mM) or NAC (20 mM). Cells were cultured at 30^ο^C for 2 days. **(D)** Addition of antioxidant GSH or NAC reduced the sensitivity of ρ^0^ cells towards SDS after salt stress. Cells were treated with 0.6 M NaCl in presence of GSH (10 mM) or NAC (20 mM) for 1 hr. Cells were washed, treated with SDS for 0.5 hr and spotted on YPD plates. Cells were then cultured at 30^ο^C for 2 days. **(E)** Diagram showing the possible involvement of *SCW11* and ROS in salt stress-induced cell wall damage and cell death of ρ^0^ cells.

Mitochondria are the main source for ROS, which play an important role in yeast apoptosis. With the lack of mitochondrial DNA, it is unclear if ρ^0^ cells produce any ROS, and if ROS are involved in salt stress response of the ρ^0 ^cells. We first examined the ROS level using dihydroethidium (DHE), a fluorescence dye that has specificity toward superoxide for detecting ROS production 16,21,22. Little ROS was detected in wild type in the absence or presence

of NaCl. In ρ^0 ^cells, however, NaCl treatment caused a significant increase of the ROS level (Fig. 3A, 3B). Addition of antioxidant GSH or NAC only slightly improved the cell survival of ρ^0 ^cells (Fig. 3C), suggesting that ROS may not directly be involved in salt stress response and cell death of the ρ^0 ^cells. We further tested if addition of the antioxidant could improve the sensitivity of ρ^0 ^cells to SDS. As shown in Fig. 3D, in the presence of GSH or NAC, ρ^0 ^cells were much less sensitive to SDS after NaCl treatment. This result suggests that ROS likely affect the salt sensitivity of ρ^0 ^cells, at least in part, via their damage to cell wall. Deletion or overexpression of *SCW11* in ρ^0 ^cells greatly affects the sensitivity of ρ^0 ^cells towards SDS after the salt stress (Fig. 2D). However, DHE staining showed that deletion or overexpression of *SCW11 *did not affect the ROS level of the ρ^0 ^cells (Fig. S1, compared to Figs. 3A, 3B). This result further suggests that ROS are not directly involved in the cell survival or cell death of ρ^0 ^cells, but rather through their damage to cell wall. Deletion of *SCW11* makes ρ^0 ^cells more resistant, while *SCW11 *overexpression makes them more sensitive, to ROS damage.

It has been reported that ROS cause damages to DNA, proteins and lipids 23, which in turn, induce apoptotic cell death 11,24, or aging 25. To our best knowledge, this is the first report that ROS induce potential cell wall damage in ρ^0 ^cells. One possibility is that the increased level of Scw11 makes the cell wall more vulnerable to ROS.

In summary, we demonstrated that the *SCW11* level was elevated in ρ^0 ^cells and the high level of Scw11 caused ρ^0 ^cells prone to cell wall damage by salt stress. The salt stress-induced ROS further damaged the cell wall, causing decreased viability and cell death of the ρ^0 ^cells (Fig. 3E).

## MATERIALS AND METHODS

### Yeast strains and media

Yeast strains are derivatives of *S. cerevisiae* W303-1A (*MATa, leu2-3/112 ura3-1 trp1-1 his3-11/15 ade2-1 can1-100*). The *scw11* deletion was introduced by PCR-mediated gene replacement 26, replacing the complete sequence of YGL028C with G418 marker. *SCW11* overexpression was constructed using the BG1805 plasmid 27. Yeast cells were routinely grown in YPD medium (1% yeast extract, 2% peptone, 2% glucose) at 30^ο^C. For overexpression of *SCW11*, cells were preincubated in BG1805 selection medium (URA minus), then transferred to YPR (1% yeast extract, 2% peptone, 2% raffinose) overnight, and then to YPG (1% yeast extract, 2% peptone, 2% galactose).

### Transmission electron microscopy (TEM)

Cells were cultured at 30^ο^C in YPD medium to early log phase, treated with 0.6 M NaCl for 1 hr. Cells were then harvested and prepared for TEM according to 28.

### Spot dilution growth assays

Cells were precultured in YPD liquid medium overnight. Cells were harvested by centrifugation, washed three times with ddH_2_O, and then resuspended in ddH_2_O. The cell density was normalized to 1×10^7^ cells/ml. A five-fold serial dilution was made and 4 µl of each dilution was spotted onto appropriate YPD plates. For YPG medium growth, cells were precultured in YPR liquid overnight, and transferred to YPG liquid for 6 hr. A five-fold serial dilution was made as above, and spotted onto appropriate YPG plates. The antioxidants glutathione (GSH, 10 mM) or N-acetyl-L-cysteine (NAC, 20 mM) were added directly to the medium.

For the SDS sensitivity test, cells were grown in YPD liquid medium to early log phase, treated with 0.6 M NaCl (0.2 M NaCl for YPG mdium) for 1 hr, then washed with ddH_2_O, resuspended in 2-[4-(2-Hydroxyethyl)-1-piperazinyl] ethanesulfonic acid (HEPES) buffer (10 mM pH 7.5), treated with 0.1% SDS for 0.5 hr, then washed one time with HEPES buffer. A five-fold serial dilution was made as above, and then spotted onto the YPD or YPG plates.

### Semi-quantitative RT-PCR 

Total RNA from yeast was extracted using RNeasy Protect Mini Kit (QIAGEN, CA). The RT-PCR and the amplification procedure were performed as in 29. Yeast actin gene (*ACT1*) was used as control, and the primers are 5’-TGTCACCAACTGGGACGATA-3’ and 5’-AACCAGCGTAAATTGGAACG-3’. Primers used for *SCW11* amplification are 5’-CCCCAACTGTCGAATTCCTA-3’ and 5’-AAAGTAGAGGTTGGCTGCGA-3’. Quantitative analysis was conducted using ImageJ software (http://rsb.info.nih.gov/ij).

### Detection of reactive oxygen species (ROS)

The detection of ROS was performed according to 15. Briefly, cells were grown to early log phase, treated with 0.8 M NaCl for 15 min, then washed with PBS for 3 times. Cells were stained with dihydroethidium (5 µM) (Sigma Chemical Co.) for 10 min in the dark, viewed with a Zeiss 710 laser scanning confocal microscope with excitation at 514 nm (Jena, Germany).

### Statistical Analysis of Data

A two-tailed t-test was used for statistical analysis. P ≤ 0.05 was considered as statistically significant.

## SUPPLEMENTAL MATERIAL

Click here for supplemental data file.

All supplemental data for this article are also available online at http://microbialcell.com/researcharticles/salt-stress-causes-cell-wall-damage-in-yeast-cells-lacking-mitochondrial-dna/.
