# Supplementary material for: Salt stress causes cell wall damage in yeast cells lacking mitochondrial DNA
Source: Microb Cell. 2014 Mar 3;1(3):94–9. doi: 10.15698/mic2014.01.131 (PMC5349227; doi:10.15698/mic2014.01.131)
Supplement: Supplementary file 1 [file mic-01-094-s01.pdf]

Supplemental information

Gao et al., Microbial Cell (2014)

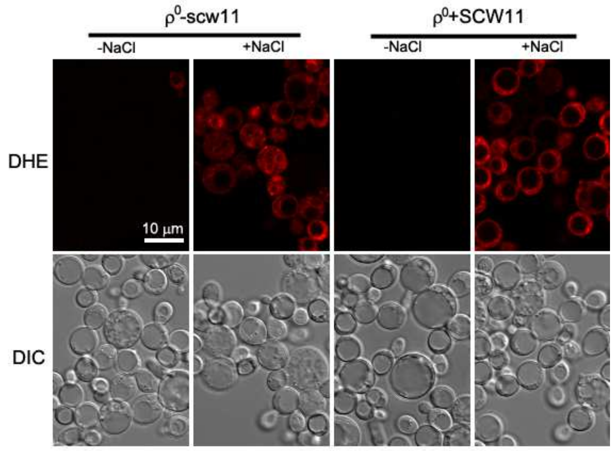

Figure S1A

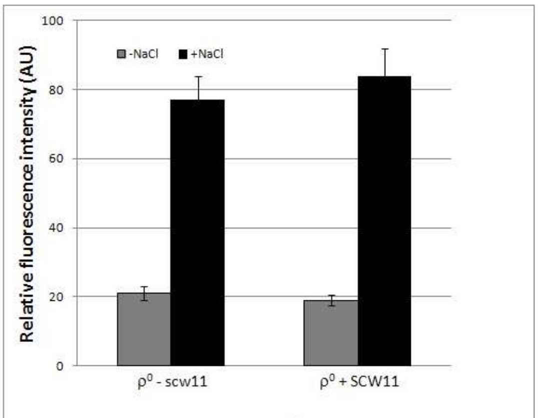

Figure S1B

Fig. S1. Deletion (ρ^0-scw11) or overexpression (ρ^0+SCW11) of SCW11 in ρ^0 cells does not affect ROS production. (**A**) Cells were treated with 0.6 M NaCl for 15 min and stained with dihydroethidium (DHE) (5 uM) after salt stress. Cells were then imaged with a Zeiss 710 confocal microscope. (**B**) Quantification of ROS production. Relative fluorescence intensities were measured by the ImageJ software. Values are means of three independent experiments. About 300 cells were measured from each experiment.
